# No risk of arthrofibrosis after acute anterior cruciate ligament reconstruction

**DOI:** 10.1007/s00167-017-4814-1

**Published:** 2017-11-29

**Authors:** Karl Eriksson, Christoffer von Essen, Sven Jönhagen, Björn Barenius

**Affiliations:** 1 Department of Orthopaedics, Stockholm South Hospital, Karolinska Institutet, Stockholm, Sweden; 2 Department of Orthopaedics, Danderyd Hospital, Karolinska Institutet, Stockholm, Sweden

**Keywords:** ACL, Acute, Outcome, Range of motion, Reconstruction

## Abstract

**Purpose:**

To compare acute ACL reconstruction (ACLR) within 8 days of injury with delayed reconstruction after normalized range of motion (ROM), 6–10 weeks after injury. It was hypothesized that acute ACL reconstruction with modern techniques is safe and can be beneficial in terms of patient-reported outcomes and range of motion.

**Methods:**

Sample size calculation indicated 64 patients would be required to find a 5° difference in ROM at 3 months. Seventy patients with high recreational activity level, Tegner level 6 or more, were randomized to acute (within 8 days) or delayed (6–10 weeks) ACLR between 2006 and 2013. During the first 3 months following surgery patients were contacted weekly by SMS and asked ‘How is your knee functioning?’, with answers given on a Visual-Analog Scale (0–10). ROM was assessed after 3 months by the rehab physiotherapist. Patient-reported outcomes, objective IKDC and manual stability measurements were collected by an independent physiotherapist not involved in the rehab at the 6-month follow-up.

**Results:**

At 3-month follow-up, 91% of the patients were assessed with no significant differences in flexion, extension or total ROM demonstrated between groups. At the 6-month follow-up, the acute group had significantly less muscle atrophy of the thigh muscle compared to the contralateral leg. Furthermore, a significantly higher proportion of patients in the acute group passed or were close to passing the one leg hop test (47 versus 21%, *p* = 0.009). No difference was found between the groups in the other clinical assessments. Additionally, no significant difference between the groups was found in terms of associated injuries.

**Conclusion:**

Acute ACLR within 8 days of injury does not appear to adversely affect ROM or result in increased stiffness in the knee joint when compared to delayed surgery.

**Level of evidence:**

II.

## Introduction

Reconstruction of the anterior cruciate ligament (ACL) following an acute rupture is commonly recommended for people wishing to return to pre-injury athletic activity [29]. In Sweden, the most common activity associated with an acute ACL rupture is soccer [16]. The incidence of ACL ruptures continues to increase, particularly amongst females [16, 18]. Patients who undergo surgery are usually younger and compete or train at a higher level than those treated non-operatively [9]. Delayed surgical reconstruction is often chosen instead of an acute reconstruction due to studies suggesting that this may reduce the risk of developing arthrofibrosis and decreased range of motion (ROM) postoperatively [6, 28]. However, in these studies, patellar bone-tendon-bone (BTB) was the preferred graft and semi-open surgical techniques were often utilized.

These techniques differ significantly from contemporary methods, with recent data showing almost 95% of all primary ACL reconstructions in the Swedish ACL-register are performed using a hamstring graft and purely arthroscopic procedures with low pressure systems are now widely used [16].

Despite these developments, early reconstruction in the first weeks following ACL rupture is still commonly avoided due to fear of postoperative stiffness, with many adhering to the theory that surgery should be postponed until swelling has subsided and the patient has regained adequate ROM. Consequently, delayed reconstruction has been recommended worldwide in clinical practice for more than 20 years.

The timing of ACLR has been discussed by Wasilewski et al. [32], and studied more recently by Bottoni et al. in a randomized controlled trial of patients undergoing ACLR with hamstring graft. In this study, no significant difference in extension or flexion loss was demonstrated when comparing surgery within 21 days of injury compared to surgery after 6 weeks [4].

Given these findings and the fact that motivated athletes with an ACL injury commonly wish to avoid unnecessary postponements to their surgery, there is a need for additional level 1 evidence confirming the safety of early reconstructive ACL surgery.

The primary aim of this study was to determine if young active patients undergoing ACLR within 1 week of injury had significantly reduced knee range of motion compared to patients undergoing reconstruction after a delay of approximately 2 months, when initial swelling and stiffness had subsided. The secondary aim was to compare early functional outcomes between the groups. It was hypothesized that an acute ACLR results in inferior patient-reported outcomes and a greater incidence of ROM deficits.

## Materials and methods

From 2006 to 2013, 2088 patients who had presented to the emergency department with an acute knee injury were followed up within 3 days at a knee clinic. Clinical examination and magnetic resonance imaging (MRI) were performed. If an ACL-rupture was diagnosed and the patient consented to participation, they were assessed for inclusion in the study.

The inclusion criteria were selected to recruit patients with a high demand for pivoting stability, thus with an obvious need for ACLR, and to exclude patients with factors that would make it difficult to follow a standardized surgical method and postoperative rehab-protocol. The inclusion criteria were: uni-lateral primary ACL-injury in patients between 18 and 40 years of age with no previous knee-injury to either leg, Tegner activity level score [31] minimum level 6, no additional meniscus or cartilage damage on MRI indicating the need for major acute meniscus or cartilage surgery, availability for reconstruction within 8 days of injury, no LCL-injury that needed surgery, no MCL-injury greater than grade 1, no PCL-insufficiency and no signs of osteoarthritis.

**Fig. 1:**
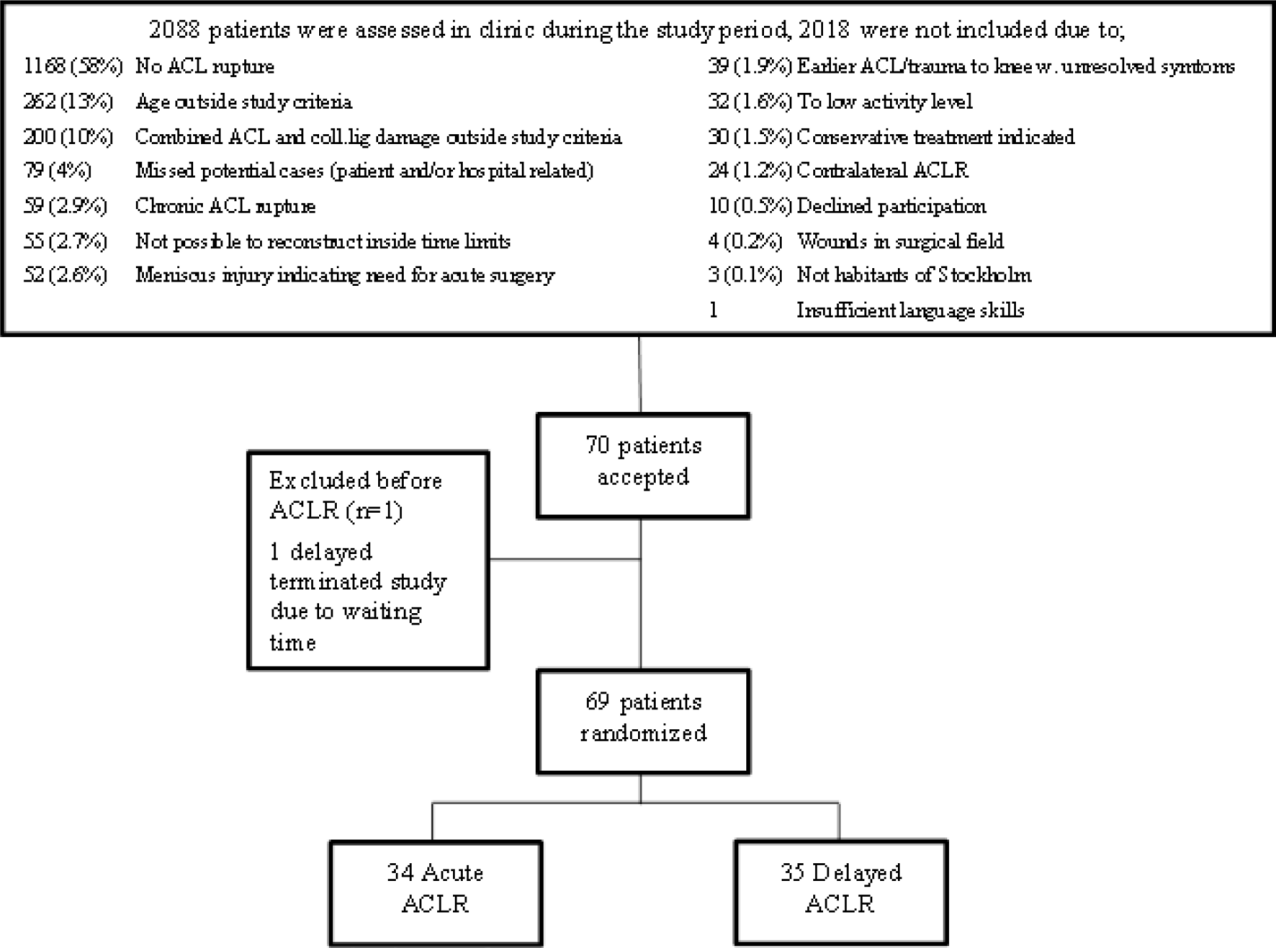
Enrollment and Randomization of Subjects

If all the pre-requisites were fulfilled, a research nurse performed randomization with the sealed envelope technique in the same session. Seventy patients were included, 35 patients were randomized to early ACLR and 35 patients to late reconstruction. One patient from the acute group dropped out before surgery due to personal reasons, a second exclusion was needed because one patient could not participate in follow-up according to the study protocol (Fig. 1). Patient demographics are presented in Table 1. The patients were prospectively randomized to reconstruction of the ACL either within 8 days of injury, or with delayed reconstruction after recovery of range of motion (ROM), between 6 and 10 weeks after the injury. The patients randomized to delayed surgery received pre-operative physiotherapy to restore normal range of movement and to preserve muscle strength.

**Table 1:** Descriptive study population

	Total (*n* = 68)	Acute ACLR (*n* = 33)	Delayed ACLR (*n* = 35)	Sign
Age at inclusion, mean ± SD	26.9 ± 6.1	27.7 ± 6.5	26.1 ± 5.7	n.s.
Gender: female, *n* (%)	21 (31)	10 (30)	11 (31)	n.s.
Length (cm), mean ± SD	177 ± 9	177 ± 9	178 ± 9	n.s.
Weight (kg), mean ± SD	77 ± 11	76 ± 11	78 ± 12	n.s.
Smoker, *n* (%)	4 (6)	2 (6)	2 (6)	n.s.
Highest education (*n* = 64) (%)				n.s.
High school/college	35 (55)	20 (65)	15 (45)	
University	29 (45)	11 (35)	18 (54)	
Main occupation, *n* (%)				n.s.
Working	51 (75)	26 (79)	25 (71)	
Student	17 (25)	7 (21)	10 (29)	
Type of injury, *n* (%)				n.s.
Soccer	26 (38)	13 (39)	13 (37)	
Indoor floorball	16 (24)	6 (18)	10 (29)	
Alpine ski/snowboard	10 (15)	7 (21)	3 (9)	
Handball	5 (7)	1 (3)	4 (11)	
Wrestling/martial arts	3 (5)	3 (9)	0	
Gymnastics	2 (3)	2 (6)	0	
Ice hockey	1 (2)	0	1 (3)	
Am. football	1 (2)	0	1 (3)	
Badminton	1 (2)	0	1 (3)	
Basketball	1 (2)	0	1 (3)	
Dance	1 (2)	1 (3)	0	
Tennis	1 (2)	0	1 (3)	

Patient demographics at baseline for patients with an ACL tear are displayed as mean ± SD, number and percentage, respectively

### Pre-operative assessments

At the time of inclusion and randomization, the patients were evaluated regarding ROM (passive ROM measured with a goniometer and reported as a deficit in extension and flexion), instrumented laxity using the Rolimeter [2] and thigh-circumference measured 10 cm proximal to the proximal pole of the patella. Subjective and self-assessed IKDC [10], KOOS [25], Lysholm and Tegner scores [31] were also evaluated.

In all clinical tests, the contralateral non-injured side was used as a reference.

### Surgical method

In each patient, an arthroscopic ACLR with autologous quadrupled semitendinosus tendon graft, or quadrupled semitendinosus- and gracilis tendon-graft if the graft was not of the defined sufficient length (minimum quadrupled graft length in this study: 6.5 cm). In the beginning of the study the tibia was drilled first and transtibial drilling of the femur was used. The tibial angle was 45°–50° as to the surgeon’s preferences. A femur entry point at 10 or 2 o’clock was preferred with a knee angle of 80–90 degrees of flexion; an offset guide of 5–7 mm was routinely used. Later on in the study due to the evolution of surgical technique at the time, we changed method for tunnel placement. Footprint positioning was used, with drilling of the femoral tunnel through an accessory medial portal, aiming for the center of the femoral footprint. The first technique included 22 patients and the last 47 patients. Fixation was standardized during the whole study period, Endobutton continuous loop^®^ (Smith & Nephew, Inc., Andover, MA 01810, USA) was used in femur and tibial fixation was performed with a metal interference-screw, RCI^®^ (Smith & Nephew, Inc., Andover, MA 01810, USA) or Soft Screw^®^ (Arthrex Inc., Naples, Florida 34108, USA). The distal fixation was reinforced with an osteo-suture over a “bone-bridge”. Distal fixation was performed in 90-degree flexion of the knee joint with subsequent testing to ensure that full extension could be attained.

For infection-prophylaxis, one intravenous dose of antibiotics (flucloxacillin) was given just prior to surgery and two extra doses after 3 and 6 h.

### Postoperative management

Full weight bearing was allowed from day 1. Antithrombotic prophylaxis with 5000 U of low molecular weight heparin was given once daily for 7 days after surgery. A brace was only used for patients who required suturing of menisci, 3 in the acute group and 1 in the delayed group. The brace had a fixed ROM 0°–60° for 4 weeks and 0–90 for another 2 weeks, full weight-bearing was permitted with the support of crutches during the first 3 weeks. Closed-chain exercises and range of motion training was initiated within 1 week of surgery. The rehabilitation was standardized to one physiotherapy- center, and the same rehabilitation protocol was used for all patients. Open-chain exercises were allowed after 6 weeks, running allowed after 14 weeks and resumption of sport activity after Biodex^®^ testing showed 90% strength in injured leg compared to the contralateral leg, but never earlier than 6 months.

### Postoperative follow-up

At 3 months ROM and circumference of the thighs were assessed by the patient’s physiotherapist. An independent physiotherapist not involved in the rehabilitation performed the same assessments at 6, 12 and 24 months.

Follow-up at 6, 12 and 24 months included the same subjective scores as preoperatively as well as functional strength test assessed with the single leg hop. Isokinetic peak torque strength at 60, 180 and 240°/s, and isometric torque strength at 60° and 180°, in both extension and flexion was measured with Biodex^®^ [30].

Follow-up included a weekly assessment to the question: “How is your knee functioning?” Answers were given on a Visual-Analog Scale (VAS) 0–10 via short message service (SMS) for the first 3 months. The question was also assessed at baseline and at 6 months together with the question “How does your knee affect your activity level?”.

The study was approved by the regional ethics committee at the Karolinska Institute, Stockholm Sweden (reference no. 2006/404-31/3/2008/1541-32).

### Statistical analysis

Statistical analysis was performed with the IBM SPSS 22.0 software package for Macintosh. Nominal variables were tested by the *χ* ^2^ test or the Fisher’s exact test. Ordinal variables and non-normality distributed interval and scale variables were evaluated by the Mann–Whitney *U* test, and the Student’s *t* test was used for normally distributed scale variables in independent groups. Longitudinal statistics were done with the paired-samples t test for normally distributed scale variables and the Wilcoxon signed-rank test for ordinal and non-normality distributed scale variables. The tests were two-sided. The results were considered significant at *p* < 0.05.

A sample size calculation was performed using the primary outcome variable ROM. If the mean difference is 5° or more (corresponding to means of 122.5 versus 117.5) and the common within-group standard deviation is 7.0. The study will have a power of 80% to yield a statistically significant result with 5% risk of a type-one error, with the proposed sample size of 32 patients for the two groups.

## Results

Demographic data of the study groups are displayed in Table 2. The only significant difference between the groups was the time between injury and reconstruction. Two patients, one in each group, were lost to follow-up at 6 months. It is also notable that mean surgery time in the acute group was longer, but not statistically significant.

**Table 2:** Demographics

		Acute ACLR (*n* = 33)	Delayed ACLR (*n* = 35)	Sign.
Time injury-recon	d ± SD	5 ± 2	55 ± 8	< 0.01
OP time	Min ± SD	93 ± 20	83 ± 18	n.s.
ST/Gr	*n* (%)	7 (21)	7(20)	n.s.
Graft diameter	Mm ± SD	8.8 ± 0.8	8.6 ± 0.8	n.s.
Additional injury	*n* (%)	21 (66)	15 (47)	n.s.
Medial meniscus	*n* (%)	7 (22)	2 (6)	n.s.
Lateral meniscus	*n* (%)	13 (41)	10 (31)	n.s.
Sutures	*n* (%)	3 (9)	1 (3)	n.s.
Cartilage inj.	*n* (%)	10 (31)	4 (13)	n.s.

Patient demographics at baseline for patients who underwent ACLR are displayed as mean ± SD, number and percentage, respectively. Statistical significant (*p* < 0.05) values were only seen for the time from injury to reconstruction

*ACL* anterior cruciate ligament reconstruction

Sixty-four patients (91%) were assessed by a non-blinded physiotherapist at 3 months. No difference in flexion, extension or total ROM between the groups was found (Table 3).

**Table 3:** ROM primary endpoint at 3 months (measured at the rehabilitation physiotherapy unit) and at 6 months (measured at the hospital unit, not part of rehab)

Degrees (SD) w ref CL limb	Acute ACLR (*n* = 32–33)	Delayed ACLR (*n* = 33–34)	Sign
3 months			
Extension, mean hyperextension	0.6 (2.2)	0.3 (1.1)	n.s.
Extension defect	3 (3.5)	2 (2.4)	n.s.
Flexion defect	7 (7.1)	6 (7.8)	n.s.
Total ROM defect	10 (9.2)	8 (8.0)	n.s.
Ext. def > 5° compared to CL, *n* (%)	10 (31)	5 (15)	n.s.
6 months			
Extension defect	3 (3.0)	4 (3.5)	n.s.
Flexion defect	4 (5.4)	5 (5.4)	n.s.
Ext. def > 5° compared to CL, *n* (%)	7 (21)	13 (37)	n.s.

Distribution of ROM between acute and delayed ACLR, displayed as mean degree defect with reference uninjured limb and SD, number and percentage, respectively

*ACL* anterior cruciate ligament reconstruction, *CL* uninjured contralateral limb

Similar ROM between the groups was found at 6 months measured at the hospital unit (Table 3).

**Fig. 2:**
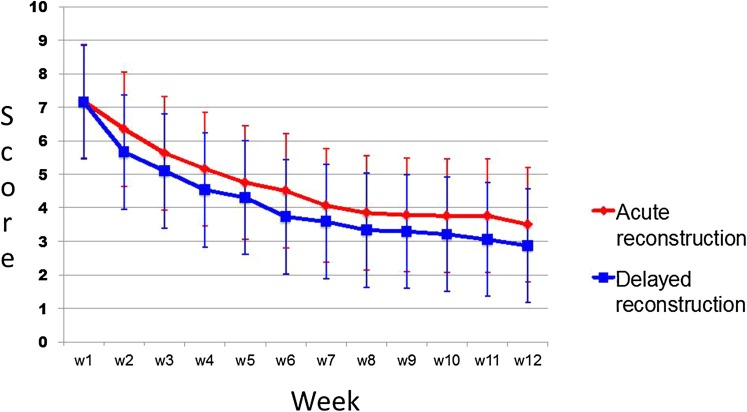
How is your knee working? Weekly SMS survey for the first 3 months after the reconstruction. The diagram above show the mean results from the SMS-survey, red lines for the acute group and blue for the delayed. The error bars indicate one standard deviation. Ten was defined as no knee function and 0 normal function. There was no significant difference between the groups at any time-point

Similar results were found in both groups for the weekly SMS question (Fig. 2) Fewer patients in the acute group reported having their activity level affected by symptoms from their knee (Table 4). Both groups had improved Tegner and Lysholm scores from inclusion to the 6-month follow-up (Table 4).

**Table 4:** Patient-reported outcomes, instrumented knee laxity and functional strength

	Acute ACLR (*n* = 32–34)	Delayed ACLR (*n* = 32–35)	*p* value
Patient-reported outcomes at 12 months			
Lysholm, mean (SD)^b^			
Inclusion	32 (21.5)	43 (26.2)	n.s.
6 months	76 (16.2)	79 (15.2)	n.s.
Tegner, median (range)^c^			
Before injury	8 (6–10)	9 (5–10)	n.s.
At inclusion	0 (0–6)^a^	0 (0)	0.001
6 months	4 (1–9)	4 (0–9)	n.s.
Instrumented knee laxity			
Rolimeter, mean mm (SD)	2.3 (1.4)	1.8 (1.2)	n.s.
Mean degrees (SD) w ref CL limb			
Extension defect	2 (2.1)	3 (3.3)	n.s.
Flexion defect	1.8 (2.2)	3.2 (3.4)	n.s.
No (%) normal			
Pivot shift test^d^	30 (94)	29 (88)	n.s.
IKDC objective score, *n* (%)			
6 months			
AB	27 (82)	24 (71)	n.s.
CD	6 (18)	10 (29)	
Functional strength			
Thigh deficit circ. 10 cm above patella diff in cm (SD) ref CL	1.0 (1.1)	1.6 (1.2)	0.04
One leg hop, *n* (%)			
> 90	15 (47)	7 (21)	0.01
76–89	11 (34)	10 (29)	
50–75	6 (19)	9 (27)	
< 50	0	8 (24)	
Muscle strength Biodex^®e^			
Ext. isokinetic			
60°/s	72	64	n.s.
180°/s	79	72	n.s.
240°/s	81	75	n.s.
Flex. isokinetic			
60°/s	85	82	n.s.
180°/s	90	82	n.s.
240°/s	94	88	n.s.
Ext. isometric			
60°	87	83	n.s.
180°	85	86	n.s.
Flex. isometric			
60°	82	77	n.s.
180°	84	75	n.s.
VAS question^f^, mean (SD)			
VAS 1			
Inclusion	83 (29)	76 (32)	n.s.
6 months	30 (24)	39 (26)	n.s.
VAS 2			
Inclusion	86 (25)	82 (29)	n.s.
6 months	39 (23)	53 (31)	0.05

*ACL* anterior cruciate ligament, *CL* uninjured contralateral limb

^a^One patient answered 6 at inclusion

^b^Score range from 0 to 100, with higher scores indicating better results

^c^Assesses activity level with specific emphasis on knee; scores range from 1 (least strenuous activity) to 10 (high knee demanding activity on professional sports level).

^d^Assesses rotational stability of knee at rest result range from 0 (normal stability) to 3 (severely increased instability)

^e^Comparison of extensor and flexor torque deficits collected for isometric Biodex, displayed as mean percentage with reference uninjured CL set at 100

^f^VAS 1 “How does your knee function (0 (normal)–100)”, VAS 2 “How does your knee affect your activity level (0 (not at all)–100)”

**Fig. 3:**
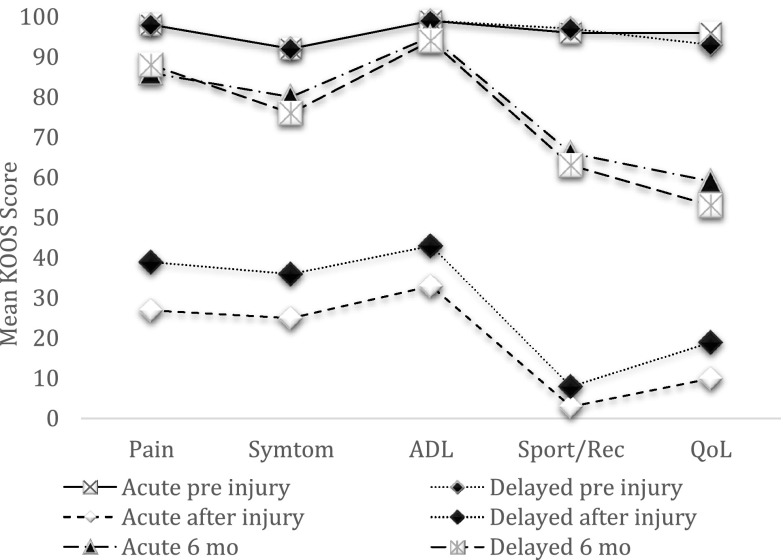
Mean KOOS score. Mean KOOS scores with significant changes after injury to 6 months, but no significant difference between the groups at any time

After the injury, the acute group were less affected in the KOOS subscales ‘pain’ and ‘quality of life’. After 6 months, the KOOS was similar in the groups, but with better improvement within the subscales ‘pain’, ‘symptoms’ and ‘quality of life’ for the acute group (Fig. 3).

The overall objective, IKDC as well as manual laxity measurements using pivot shift and rolimeter did not display any statistically significant differences (Table 4).

There was less muscle atrophy and more patients with a normal one leg hop test in the acute group (Table 4). However, no differences between the groups in peak torque was found during the Biodex^®^ test (Table 4).

With a cutoff for return to sport at 90% or more strength in the reconstructed limb compared to the contralateral limb in extension and flexion at 60°/s, 4 of 33 acute and 2 of 34 delayed patients were cleared at 6 months.

## Discussion

The most important finding of this randomized control trial is that at 6-month follow-up there were no significant differences in ROM between patients who underwent acute ACLR compared to patients undergoing delayed surgery. Furthermore, there were no significant differences in most of the subjective outcome scores measured at 6-month follow-up. This finding challenges the current “state of the art” regarding timing of ACLR, that acute reconstruction should be avoided due to increased risk of stiffness.

The landmark study by Shelbourne et al [28] is the primary source for the recommendation to delay ACLR by at least 3 weeks. Other studies have found similar results [23, 32], however, these studies were retrospective, included patients with additional ligament injuries and did not use contemporary arthroscopic techniques. Importantly, these studies also had a restrictive postoperative rehabilitation regime. Shelbourne et al [26, 27] reported significantly less arthrofibrosis when an accelerated rehabilitation program was used.

Mayr et al [20] retrospectively reviewed a cohort of 156 patients with post-operative arthrofibrosis and found significant correlations between knee irritation, effusion and swelling and development of arthrofibrosis, rather than the time from injury to surgery. The rationale for acute reconstruction is that if the surgery is performed within the first few days after the injury, the surgical trauma itself will blend into the trauma from the injury. In contrast, a slightly delayed operation when healing is already underway may result in a second hit, due to surgical trauma, resulting in an increased risk for arthrofibrosis. In our study, acute reconstruction did not result in increased stiffness. The acute group had better one leg hop tests, better improvements within the subscales pain, symptoms and quality of life in KOOS, and better outcomes regarding how the knee affected their activity level at 6 months. Additionally, the acute group was not inferior to the delayed group in any assessment. These findings are supported by the systematic review by Andernord et al [1].

The timing of surgery may also affect other important outcomes such as; occurrence of additional injuries (predominantly cartilage and meniscus), development of muscle wasting, final outcome after surgical treatment, time between injury and return to play as well as patient satisfaction. In a study from 1995, early reconstruction with patellar tendon graft, or fascia lata graft, was compared to delayed reconstruction. Patients with an early reconstruction returned to sports activities sooner and had better clinical results [19].

Meighan et al. reported that there was no advantage in early reconstruction within 2 weeks of the injury, and a higher rate of complications [21]. However, more recent studies support early reconstruction. Bottoni et al. reported excellent results after reconstruction within 3 weeks of injury, with no subjective or clinical differences in ROM. Their results are more in line with the findings in the present study [4]. Herbst et al. compared acute ACLR within 48 h after injury with delayed reconstruction and stated that the outcome of an ACLR does not depend on surgical timing [11]. In addition, the likelihood of normalized knee kinematics has been shown to correlate with time between injury and reconstruction [12–14].

Other studies have examined whether surgery should be delayed to see if a patient can successfully be managed conservatively. Frobell et al. [7, 8] concluded that early reconstruction was not superior to initial nonsurgical treatment with optional delayed reconstruction, however at 5-year follow-up 51% in the nonsurgical group had undergone delayed reconstruction. The odds of having a meniscus lesion significantly increase, as the time between injury to surgery increase [5, 22] and there are reports of a higher prevalence of OA with longer time between injury and reconstruction [15, 17]. This raises the question of whether initial nonsurgical treatment in patients with a high pre-injury activity level is an acceptable option. We did not find any significant difference between the groups in terms of associated injuries; a finding supported by other studies [4, 21, 24], though differences in the development of associated injuries have been seen in larger cohorts [3]. It is possible that no difference was detected in our study due to the smaller cohort size, or due to the difference in time-to-surgery between the groups being too small to influence additional injury outcomes.

The major strength of this study is the prospective, randomized design with four experienced surgeons performing all of the ACLRs with the same surgical technique. Furthermore, one center with the same postoperative rehabilitation protocol was used in both groups. The two groups were also comparable in terms of age, gender and pre-injury Tegner activity lever, factors which could contribute to selection bias in a non-randomized trial.

Potential limitations are the limited number of patients, though there were sufficient numbers according to the power analysis, and the change in surgical method during the study period (transtibial versus femoral portal drilling).

## Conclusion

In this study, acute ACLR within 8 days did not result in reduced ROM compared to delayed surgery. The patients who underwent acute reconstruction had significantly less muscle hypotrophy in the early phase of the rehabilitation and significantly better one leg hop test. No difference was found between the groups in the other clinical assessments. This study provides further evidence that acute ACL reconstruction can be performed safely without an increased risk of developing stiffness. Thus, clinicians can make their decision about the optimal time for surgery for each individual patient based on other parameters if reconstruction is planned as the primary treatment after an acute injury.
